# Application of the Improved POA-RF Model in Predicting the Strength and Energy Absorption Property of a Novel Aseismic Rubber-Concrete Material

**DOI:** 10.3390/ma16031286

**Published:** 2023-02-02

**Authors:** Xiancheng Mei, Zhen Cui, Qian Sheng, Jian Zhou, Chuanqi Li

**Affiliations:** 1 Institute of Rock and Soil Mechanics, Chinese Academy of Sciences, Wuhan 430071, China; 2 University of Chinese Academy of Sciences, Beijing 100049, China; 3 School of Resources and Safety Engineering, Central South University, Changsha 410083, China; 4 Laboratory 3SR, CNRS UMR 5521, Grenoble Alpes University, 38000 Grenoble, France

**Keywords:** aseismic concrete material, strength, energy absorption property, improved POA algorithm, tunnel

## Abstract

The application of aseismic materials in foundation engineering structures is an inevitable trend and research hotspot of earthquake resistance, especially in tunnel engineering. In this study, the pelican optimization algorithm (POA) is improved using the Latin hypercube sampling (LHS) method and the Chaotic mapping (CM) method to optimize the random forest (RF) model for predicting the aseismic performance of a novel aseismic rubber-concrete material. Seventy uniaxial compression tests and seventy impact tests were conducted to quantify this aseismic material performance, i.e., strength and energy absorption properties and four other artificial intelligence models were generated to compare the predictive performance with the proposed hybrid RF models. The performance evaluation results showed that the LHSPOA-RF model has the best prediction performance among all the models for predicting the strength and energy absorption property of this novel aseismic concrete material in both the training and testing phases (R^2^: 0.9800 and 0.9108, VAF: 98.0005% and 91.0880%, RMSE: 0.7057 and 1.9128, MAE: 0.4461 and 0.7364; R^2^: 0.9857 and 0.9065, VAF: 98.5909% and 91.3652%, RMSE: 0.5781 and 1.8814, MAE: 0.4233 and 0.9913). In addition, the sensitive analysis results indicated that the rubber and cement are the most important parameters for predicting the strength and energy absorption properties, respectively. Accordingly, the improved POA-RF model not only is proven as an effective method to predict the strength and energy absorption properties of aseismic materials, but also this hybrid model provides a new idea for assessing other aseismic performances in the field of tunnel engineering.

## 1. Introduction

The research and development of aseismic concrete materials is of great significance to reducing the damage caused by earthquakes on tunnel structures. To determine the material’s performance and decide its application in the field of tunnel engineering, the strength and energy absorption properties are widely used as the main performance evaluation indices [1,2,3,4]. Among numerous novel concrete materials and various component combinations, rubber-concrete material has received much attention for its outstanding capabilities in two performance indices mentioned above [5,6]. Meanwhile, the combination of rubber and traditional concrete material is an effective approach to solving waste rubber pollution [7].

Reviewing the published research, the experiment tests are direct methods to calculate the strength and energy absorption properties of the rubber-concrete material [8]. The strength can be calculated by using the universal testing machine and the Split Hopkinson Pressure Bar (SHPB) device is usually used to measure the energy absorption property [9,10]. Nevertheless, elaborate test sample production and complex test programs are extremely time-consuming [11]. The design and construction of short duration tunnels may be delayed due to time loss, resulting in an inability to resist earthquake shocks.

In recent years, soft computing represented by machine learning (ML) has made remarkable achievements in the prediction of rubber-concrete material performance, e.g., artificial neural networks (ANN) [12,13], support vector machine (SVM) [14,15], back-propagation neural network (BPNN) [16,17], extreme learning machine (ELM) [18,19], multi-layer perceptron (MLP) [20,21] and trees-based models [22,23]. Among the ML models, the random forest (RF) model has an excellent resistance to overfitting and a fitting ability in solving prediction problems [24]. Farooq et al. [25] proposed an optimized RF model using the gene expression programming (GEP) to forecast the strength of high strength concretes. This model has obtained a better performance than the decision tree model and artificial neural networks. Mai et al. [26] utilized the RF model to determine the strength of a novel concrete material, which contains ground granulated blast furnace slag. The prediction results showed that the RF model is a more suitable predictor than time-consuming experiments for engineering. However, the RF model is rarely used to predict the aseismic performance of rubber-concrete materials. Sun et al. [27] used the RF model to predict rubber concrete’s strength, which obtained a good predictive performance, the correlation coefficient of which (R) was 0.9596 and the root mean square error (RMSE) was 3.9032. In addition, the energy absorption property of the rubber-concrete material has not been investigated using soft techniques, especially the ML models. Furthermore, numerous metaheuristic optimization algorithms based on swarm intelligence (SI) are used to further improve the predictive ability of ML models for predicting the aseismic concrete material’s performance, e.g., particle swarm optimization [28], grey wolf optimizers [29], sparrow search algorithms [11], artificial bee colonies [30] and firefly algorithms [31]. In addition, building more reasonable initial populations of SI algorithms is an effective means to strengthen their optimization abilities [32].

Therefore, an optimized RF model using the pelican optimization algorithm (POA) is proposed to predict the strength and energy absorption properties of a novel aseismic rubber-concrete material in this study. Two functions named Latin hypercube sampling (LHS) and Chaotic mapping (CM) are adopted to improve the initial population of POA. In total, 140 concrete specimens are generated to test their strength (70 specimens) and energy absorption property (70 specimens) for training the prediction models. Four statistical indices are utilized to evaluate the predictive performance of the improved POA-based RF model and other comparison models. Finally, sensitive analysis is responsible for calculating the parameters’ importance in the strength and energy absorption property prediction.

## 2. Research Significance

As a new functional material with special uses, rubber-concrete material is mainly aimed at weakening the dynamic impact of engineering structures under strong earthquake disturbance. The incorporation of rubber means more impact energy is absorbed and converted into other forms of energy removal, but also leads to the reduction of the rubber concrete’s strength. A traditional uniaxial compression test and an impact test based on the SHPB device are the main technical approaches to measure the strength and energy absorption properties of aseismic materials. Although the test results are accurate, sample preparation and equipment operation are extremely time consuming. In the face of urgent engineering needs, the performance estimation of seismic materials without timely estimation is bound to delay the construction period and bring certain economic losses. Moreover, ordinary experimental results and regression analysis cannot accurately grasp the nonlinear relationship between material properties, material proportions, geometric properties and material performance. Therefore, the development of a novel performance estimation method is of great significance for the close fit between novel seismic materials and practical engineering.

Soft computing represented by ML models may be used to learn this nonlinear relationship and make fast and accurate predictions of a material’s performance. This study contributes (a) to the development of a novel aseismic concrete material consisting of rubber, sand and concrete and (b) to the accurate prediction of the strength and energy absorption properties of aseismic material, which is significant to get rid of the miscellaneous material performance tests based on large-scale equipment and to measure them in real time in field engineering. The improved POA-RF model is provided to estimate the strength and energy absorption properties of a novel aseismic rubber-concrete material using an actual database collected from a series of performance tests in this study.

## 3. Experiment of a Novel Aseismic Concrete Material

To enhance tunnel stability under earthquake resistance, a novel aseismic concrete material doped with rubber and river sand is developed and its performance is assessed. The rubber is responsible for increasing the elasticity and damping of the novel aseismic material, and the river sand is utilized to provide enough strength for it. Although some novel fine aggregates were put forward instead of river sand, e.g., blast furnace slag [33], waste glass [34] and plastic box waste [35], low workability and some substandard physical properties (e.g., voids and abrasion) make them difficult to be adopted in practical projects [36]. Therefore, river sand is still considered the ideal fine aggregate for composing the novel concrete material in this work.

**Figure 1:**
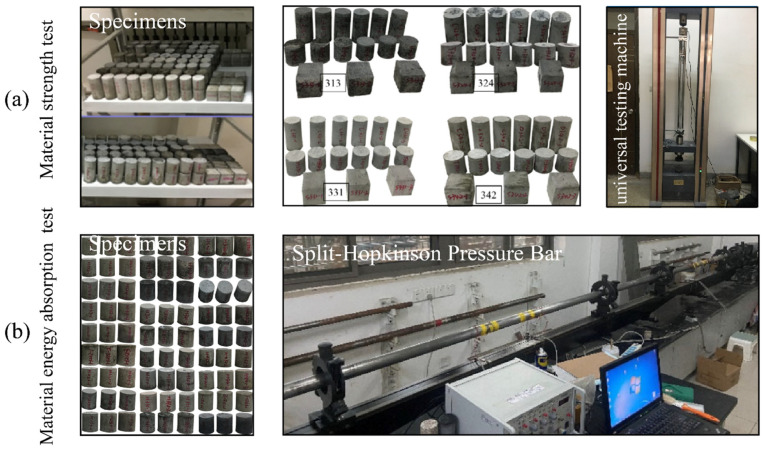
The performance test diagrams of the novel aseismic rubber-concrete material.

Before testing the strength and energy absorption properties of a novel aseismic rubber-concrete material, the concrete specimens should be prepared according to the test standards, and the production procedure is shown in Table 1. As illustrated in Table 2, there are 27 mix proportions designed for the rubber-concrete material to test the strength (cylindrical shape) and energy absorption properties (disc shape). The rubber mass ratios ($RM=M_{rubber}/M_{RSM}$), where $M_{rubber}$ is the rubber mass and $M_{RSM}$ is the rubber-sand mixtures (RSM) mass, are set to 0, 10, 30, 50, 70 and 100% and four different ranges of rubber particle size (RPS) are selected. Thus, the sand mass ratios ($SM=M_{sand}/M_{RSM}$), where $M_{sand}$ is the sand mass, are set to 0, 30, 50, 70, 90 and 100%. The cement mass ratios ($CM=M_{cement}/M_{RC}$), where $M_{cement}$ is the cement mass and $M_{RC}$ is the specimen mass, are selected as 30, 40, 50 and 60%. Three specimens are prepared for each mix proportion. Because of the influence of test operation, mechanical failure and other factors, the accuracy of some test data is suspicious. As a result, a total of 140 concrete specimens are concreted to carry out experimental tests. Seventy specimens are used to calculate the material strength using the universal testing machine and the rest of specimens are tested using SHPB device to obtain the energy absorption property as shown in Figure 1. To accurately describe the aseismic performance of this novel rubber-concrete material, the uniaxial compressive strength (UCS) and the energy transmission rate (ETR) are adopted to quantify the strength and the energy absorption property, respectively [37]. The UCS and the ETR can be defined using Equations (1) and (2), respectively.

(1) $$\delta_{specimen}=P_{\max}/S_{specimen}$$

(2) $$\begin{array}{l} E_{I}(t)=EM_{bar}\cdot \nu \cdot S_{bar}\int_{0}^{t}\varepsilon_{I}{}^{2}(t)dt\\ E_{T}(t)=EM_{bar}\cdot \nu \cdot S_{bar}\int_{0}^{t}\varepsilon_{T}{}^{2}(t)dt \end{array}$$

where $\delta_{specimen}$ and $S_{specimen}$ represent the UCS and the compression area of the tested specimen. $P_{\max}$ is the maximum collapse load. $E_{I}$ and $\varepsilon_{I}$ represent the incident energy and the measured strains on the incident bar, respectively. $E_{T}$ and $\varepsilon_{T}$ indicate the transmitted energy and the measured strains on the transmission bar, respectively. For the SHPB bar, $EM_{bar}$ is the elasticity modulus, ν is the stress wave velocity and $S_{bar}$ is the cross-sectional area.

**Figure 2:**
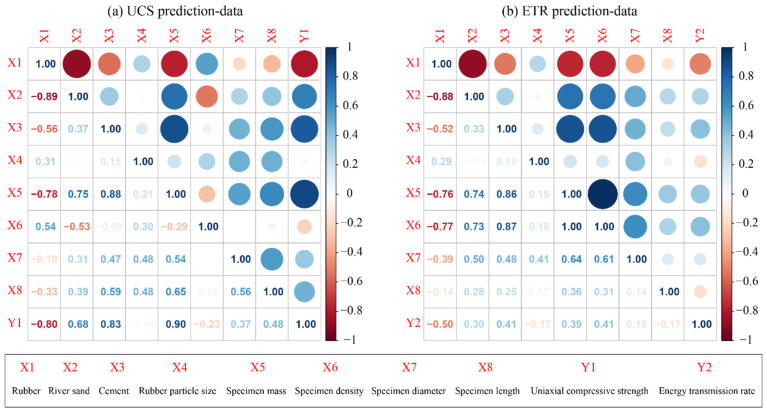
The demonstration of parameter correlation analysis: (**a**) UCS prediction and (**b**) ETR prediction.

It should be noted that each specimen is independent and non-repetitive, such as composition content (rubber particle with different size, river sand and cement), physical properties (mass and density) and size (diameter and length). The detailed information of the considered parameters is listed in Table 3 and Table 4. Nevertheless, not all parameters are favorable for predicting the UCS and ETR of the novel concrete material. The results of parameter correlation analysis are shown in Figure 2. As illustrated in Figure 2a, the correlation between rubber, river sand, cement and the specimen mass is significantly higher than that between other parameters and is close to 1, respectively. Thus, the specimen mass (M) should be removed to reduce the calculation time for predicting the UCS. On the other hand, the correlation between the M and specimen density (r) and most other parameters is not low, especially the rubber (R), river sand (S) and cement (C) for predicting the ETR (see Figure 2b). In addition, the R and S are negatively correlated, and the correlation values are high in both the UCS prediction and the ETR prediction data sets, but the reason for this result is that the rubber and river sand are taken as important components of this novel material in the form of combination. For example, concrete with low rubber content may lead to insufficient elasticity and require more river sand to increase its strength. Therefore, neither the rubber nor river sand can be deleted in this study.

## 4. Methodologies

### 4.1. Random Forest

Random forest (RF) is generally regarded as an excellent integrated machine learning model for solving classification and prediction problems. The significant advantage of this model is the data utilization and performance evaluation mechanism, i.e., bootstrap resampling technology is responsible for randomly selecting most data from the original database (the ratio is two to three) to form the decision trees (DTs) and the rest of data is utilized to establish the test set for evaluating the DTs performance; the final performance of the RF model is determined by using the average value of the predicted values of all DTs.

**Figure 3:**
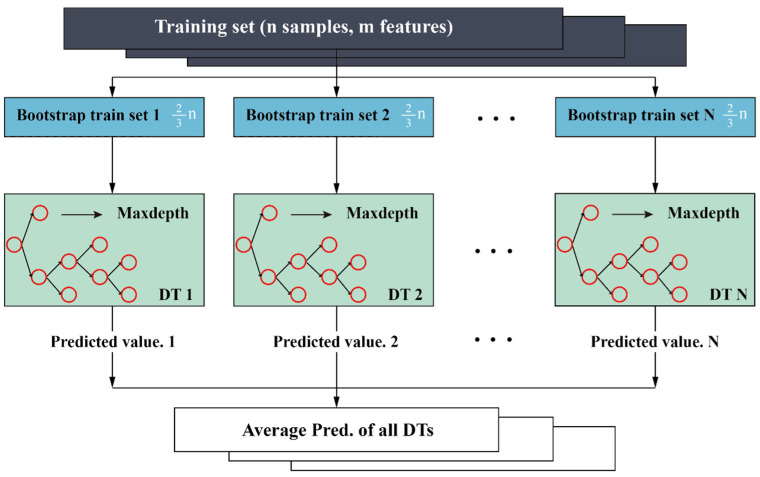
The common structure of the RF model in the regression problem.

As illustrated in Figure 3, the number of trees (N_t_) and the random features (Maxdepth) are the main hyperparameters that affect its prediction performance. Although the increase of the N_t_ will not cause the overfitting of the model, it is difficult to obtain satisfactory predictive performance through the time-consuming manual debugging of hyperparameters’ combinations [38,39].

### 4.2. Improved Pelican Optimization Algorithm

#### 4.2.1. Pelican Optimization Algorithm

Trojovský and Dehghani [40] proposed a novel metaheuristic optimization algorithms-based swarm intelligence to solve the optimization problem, that is, the pelican optimization algorithm (POA). The development of the algorithm was inspired by the fact that pelicans often hunt prey such as fish in groups. Pelicans’ hunting behavior is full of wisdom. For example, pelicans determine the position of prey in advance, and then approach the prey quickly and complete the hunting behavior with a swotting posture when the distance between them is close to 10–20 m [41]. The detailed framework of the POA can be represented using following steps:

(a) Population initialization: Pelicans generally search for prey within a certain range of search space, so the initial position of each pelican in the population is random and can be expressed as:

(3) $$P_{i}=S_{\min}+rand\cdot (S_{\max}-S_{\min})i=1,\ldots ,\;I$$

where $P_{i}$ represents the initial position of the *i*-th pelican. *I* indicates the maximum number of pelicans. $S_{\min}$ and $S_{\max}$ are the minimum and the maximum boundaries of the search space, respectively. rand⋅() represents a random number in (0, 1).

(b) Exploration phase: At this stage, the main goal of pelicans is to find and determine the prey position and change their positions in preparation for an attack. Therefore, the position of each pelican is updated using Equation (4):

(4) $$P_{i}^{1}=\left\{ \begin{array}{l} P_{i}+rand\cdot (P_{p}-U\cdot P_{i}),f_{p}<f_{i}\\ P_{i}+rand\cdot (P_{i}-P_{p}),else \end{array}\right.$$

where $P_{i}^{1}$ and $P_{p}$ represent the updated position of the pelican and the prey position in the exploration phase, respectively. $f_{p}$ and $f_{i}$ indicate the objective functions of the prey and the pelican, respectively. *U* is also a random number (1 or 2).

(c) Exploitation phase: Once the pelican is in a good position and launches an attack, i.e., flying across the water and pushing the fish up into its throat pouch. This strategy can be mathematically expressed as:

(5) $$P_{i}^{2}=P_{i}+z\cdot \left(1-t/T\right)\cdot \left(2\cdot rand-1\right)\cdot P_{i}$$

where $P_{i}^{2}$ represents the updated position of the pelican in the exploitation phase. *z* is a random number (0 or 2). *T* is the maximum number of iterations, and *t* represents the current iteration.

#### 4.2.2. Optimization Methods

For the metaheuristic optimization algorithms-based SI, the incompatibility of population initialization results in low precision and an easy to fall into local minimum problem [42]. Numerous researchers have utilized various strategies to optimize the population initialization of SI algorithms, e.g., opposition-based learning [43,44], Gaussian mutation [45,46], normal distribution [47] and multi-subgroup [48]. In addition, the chaotic mapping (CM) and the Latin hypercube sampling (LHS) have also widely been used to improve the performance of the SI algorithms by adjusting the population initialization [49,50]. The former is characterized by traversal and random, while the latter has significant space-filling impact and convergence features for solving the population initialization problem [51,52]. Therefore, CM and the LHS are adopted to improve the POA for predicting the UCS and the ETR of a novel aseismic rubber-concrete material in this study.

##### Chaotic Mapping Method (CM)

The aim of the CM method is to use mapping functions to generate a more diverse population of the POA. The logistic mapping function is a popular method to achieve this goal [53,54], which can be expressed using Equation (6).

(6) $$Log^{i+1}=\lambda Log^{i}(1-Log^{i})0\leq \lambda \leq 4$$

where $Log^{i}$ and $Log^{i+1}$ represent the *i*-th and *i*+1-th chaotic sequence of the logistic mapping function, respectively. λ is a constant. Thus, Equation (3) can be rewritten as:

(7) $$P_{i}^{\log}=P_{i}\times (1-\eta )+\eta f_{\log},i=1,2,\ldots ,I$$

where $P_{i}^{\log}$ represents the new initial position of the *i*-th pelican. η indicates an iteration factor. $f_{\log}$ is the logistic mapping function.

##### Latin Hypercube Sampling Method (LHS)

The function of the LHS is to stratify the initial positions in three-dimensional space, and then conduct random sampling and disrupt the order of each sample to obtain a more stable and diverse population. Therefore, the new population can be described as follows:

(8) $$P_{i}^{LHS}={\left[\begin{array}{c} P_{1}\\ \vdots \\ P_{i}\\ \vdots \\ P_{I} \end{array}\right]}_{I\cdot K}={\left[\begin{array}{ccccc} P_{1,1} & \cdots & P_{1,k} & \cdots & P_{1,K}\\ \vdots & \ddots & \vdots & & \vdots \\ P_{i,1} & \cdots & P_{i,k} & \cdots & P_{i,K}\\ \vdots & & \vdots & \ddots & \vdots \\ P_{I,1} & \cdots & P_{I,k} & \cdots & P_{I,K} \end{array}\right]}_{I\cdot K}$$

where $P_{i}^{LHS}$ represents the new initial position of the *i*-th pelican. *k* and *K* indicate the current dimension of pelican and the maximum dimension of the search space, respectively.

### 4.3. A Novel Combination of the IPOA and RF Model

**Figure 4:**
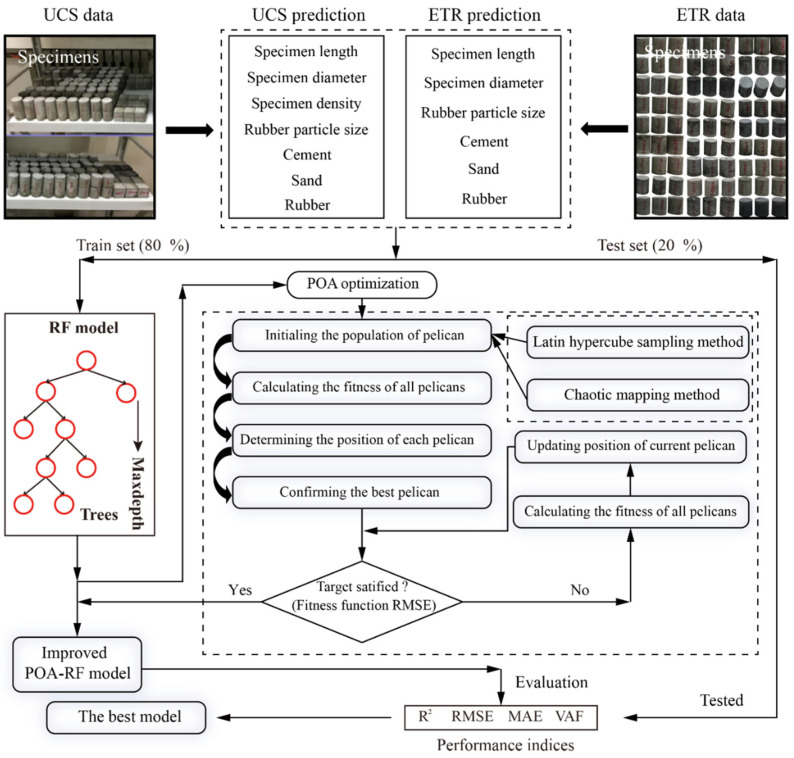
The framework of predicting the UCS and ETR of the novel concrete material.

Two improved optimization algorithms consisting of Latin hypercube sampling-POA (LHSPOA) and chaotic mapping-POA (CMPOA) are used to optimize the RF model for predicting the UCS and the ETR of a novel aseismic rubber-concrete material in this study. As illustrated in Figure 4, the framework of using the proposed models to predict the aseismic performance of the novel concrete material can be organed in three parts: (a) Building the database—as mentioned in Section 3, 70 samples were used to predict the UCS of this novel aseismic concrete material, and another 70 samples were used to predict the ETR. Whether the UCS or the ETR prediction, the ratio of the train set to the test set is four to one. It should be noted that all data is normalized to [−1, 1] to eliminate the data discrepancies; (b) Developing prediction models—the POA and the improved POA were combined with an RF model to generate different prediction models, i.e., POA-RF, LHSPOA-RF and CMPOA-RF. The population size is a key factor affecting the optimization algorithm’s performance [55,56], which is set as 20, 40, 60, 80 and 100 to explore the potential of the model. The iteration is 200 and the RMSE is considered as a fitness function to determine the optimal population size. For the RF model, the ranges of N_t_ and Maxdepth are (1, 100) and (1, 10), respectively; (c) Performance evaluation—four statistical indices were used to evaluate the predictive performance of the proposed hybrid RF models.

## 5. Performance Evaluation

In this study, four indices were used to evaluate the performance of the proposed models for predicting the UCS and the ETR of the novel aseismic concrete material. The determination coefficient (R^2^) is also known as goodness of fit, which reflects the interpretation of the predicted value to the measured value from the fitting perspective. If R^2^ is equal to 1, that means the prediction model is perfect. The variance accounted for (VAF) is often used to evaluate the degree to which the prediction model can explain the variance of the considered data. The function of the root mean square error (RMSE) is to evaluate the model performance by measuring the error between the predicted value and the measured value. In addition, the mean absolute error (MAE) can further reflect the real situation of error. These statistical indices have been considered to verify the performance of different prediction models for solving the regression problem [57,58,59,60,61,62].

(9) $$R^{2}=1-\frac{{\left[\sum_{t=1}^{T}\left(m_{t}-p_{t}\right)\right]}^{2}}{{\left[\sum_{t=1}^{T}\left(m_{t}-\bar{m}\right)\right]}^{2}}$$

(10) $$\mathrm{VAF}=\left[1-\frac{\mathrm{var}(m_{t}-p_{t})}{\mathrm{var}(m_{t})}\right]\times 100$$

(11) $$\mathrm{RMSE}=\sqrt{\frac{1}{T}\sum_{t=1}^{T}{\left(m_{t}-p_{t}\right)}^{2}}$$

(12) $$\mathrm{MAE}=\frac{1}{T}\sum_{t=1}^{T}\left|m_{t}-p_{t}\right|$$

where *T* indicates the number of used samples. *m_t_* and *p_t_* represent the *t*-th measured and predicted values, respectively. $\bar{m}$ is the average of the measured values.

## 6. Results and Discussion

### 6.1. The Development Results of the Proposed Models

**Figure 5:**
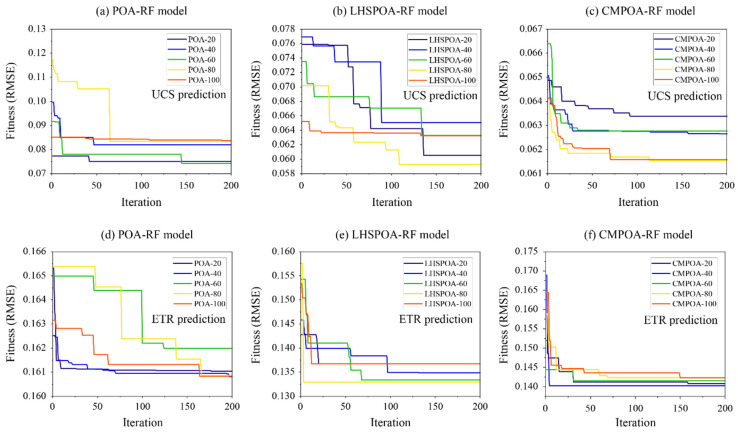
The development results of the proposed models for predicting the UCS and ETR of the novel aseismic concrete material.

To determine the best population size of each prediction model, the fitness values of each model with five types of population are calculated during 200 iterations. The development results of the proposed models for predicting the UCS of the novel aseismic concrete material are shown in Figure 5a. The best population size of the POA-RF model is 60 by means of the lowest value of the fitness. The LHSPOA-RF model and the CMPOA-RF model obtained the lowest values of fitness when the numbers of the population size are both 80, as shown in Figure 5b,c. However, it should be noted that the fitness values of the best LHSPOA-RF model and the best CMPOA-RF model are lower than the POA-RF model (see Table 5). As illustrated in Figure 5d, the POA-RF model with a population size of 40 achieved a lower fitness value than other models for predicting the ETR of the novel aseismic concrete material. Compared with the best POA-RF model, the most satisfying population sizes of the LHSPOA-RF model and the CMPOA-RF model are 80 and 40, respectively (see Figure 5e,f). As a result, the optimal hyperparameter combinations of RF models based on the three optimization algorithms for predicting the UCS and the ETR are listed in Table 5.

### 6.2. Performance Evaluation for the UCS and the ETR Prediction

After establishing the optimal hybrid RF models, each model was first utilized to predict the UCS and the ETR of this novel aseismic concrete material based on the train set. The performance indices of all the models are listed in Table 6. As can be observed in this table, the LHSPOA-RF model is not only the best model with the highest accuracy (R^2^: 0.9800, RMSE: 0.7057, MAE: 0.4461 and VAF: 98.0005%) for predicting the UCS, but also obtained better performance indices (R^2^: 0.9108, RMSE: 1.9128, MAE: 0.7364 and VAF: 91.0880%) than other models in the ETR prediction. After this model, the performance indices of the CMPOA-RF model are also better than the POA-RF for predicting the UCS and ETR.

**Figure 6:**
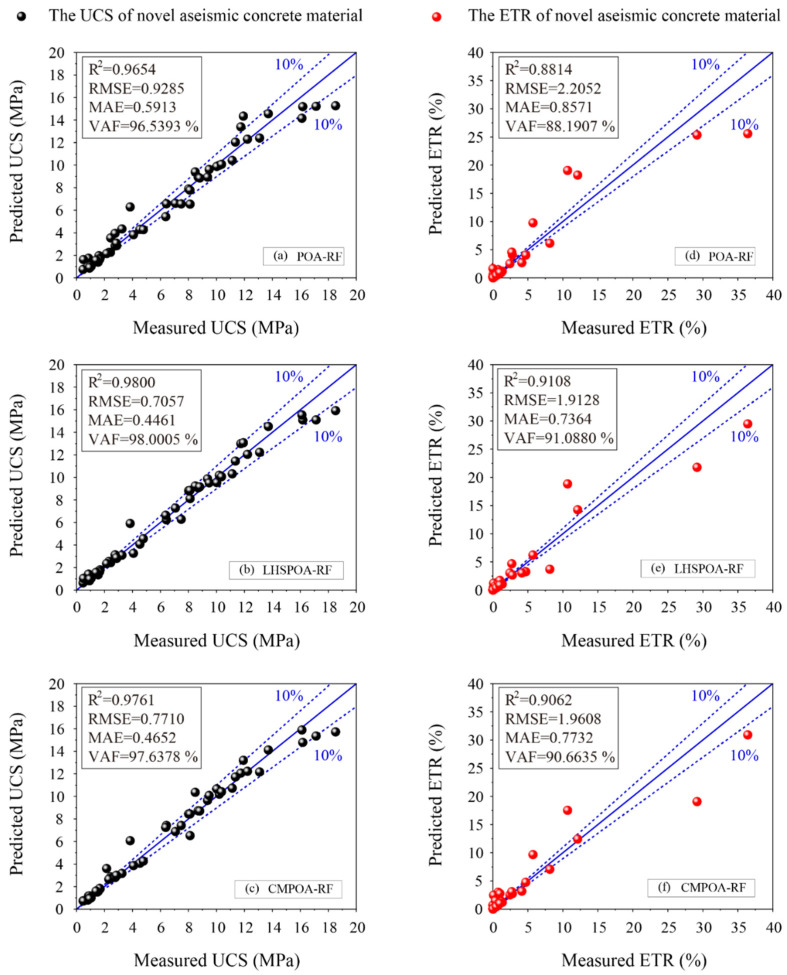
The regression diagrams of the proposed models for predicting the UCS and ETR in the training phase.

In addition, the rank score is a useful tool to evaluate the model performance by calculating the score of all the performance indices. For example, the score values corresponding to the highest values of R^2^ and RMSE in the same set are 4 and 1, respectively. As demonstrated in Table 6, the LHSPOA-RF model has achieved the highest values of ranking scores (12 and 12) for predicting the UCS and the ETR in the training phase. Figure 6 illustrates the regression diagrams of the proposed hybrid RF models using the train set. In each diagram, the blue line represents the perfect prediction function y = x, i.e., the predicted value is equal to the measured value. Therefore, the more points that locate on the blue line or close to it, the more superior the model’s performance compared with others. Of course, the regression lines with 10% have similar functions for evaluating the models’ performance. As can be seen in Figure 6b,e, the LHSPOA-RF model not only obtained the most data points close to or located at the blue line in the UCS prediction, but also showed the same excellent prediction performance in the ETR prediction resulting in the best performance indices. After the LHSPOA-RF, the CMPOA-RF model has achieved a more satisfying predictive performance than the POA-RF model resulting in more data points within the 10 % lines for the UCS prediction as shown in Figure 6a,c, and obtained the same performance for predicting the ETR (see Figure 6d,f).

However, the performance evaluation of the model in the training phase is not enough to prove its real prediction performance, especially as it is applied to predict unknown data. Therefore, the test set is responsible for verifying the performance of the proposed models for predicting the UCS and the ETR. Table 7 shows the performance evaluation results of all the models in the testing phase. Compared with the performance results obtained using the ETR dataset in the training phase, the performance indices of each model are somewhat worse, but not by much. The result indicates that the adverse overfitting phenomenon does not appear in the proposed models, which suggests that the proposed model is accepted and may be applied to practical engineering. Furthermore, the LHSPOA-RF model is still the best prediction model for forecasting the UCS and the ETR resulting in the highest values of R^2^ (0.9857 and 0.9065) and VAF (98.5909% and 91.3652%), and the lowest values of RMSE (0.5781 and 1.8814) and MAE (0.4233 and 0.9913). After the LHSPOA-RF model, the CMPOA-RF model has better performance indices than the POA-RF model for predicting the UCS and the ETR.

The ranking score results of the proposed models for predicting the UCS and ETR in the testing phase are also shown Table 7. Based on these results, the order of the three hybrid RF models is the LHSPOA-RF model, the CMPOA-RF model and the POA-RF model in the UCS prediction. The same ranking score results of each model is obtained by using the ETR test data.

**Figure 7:**
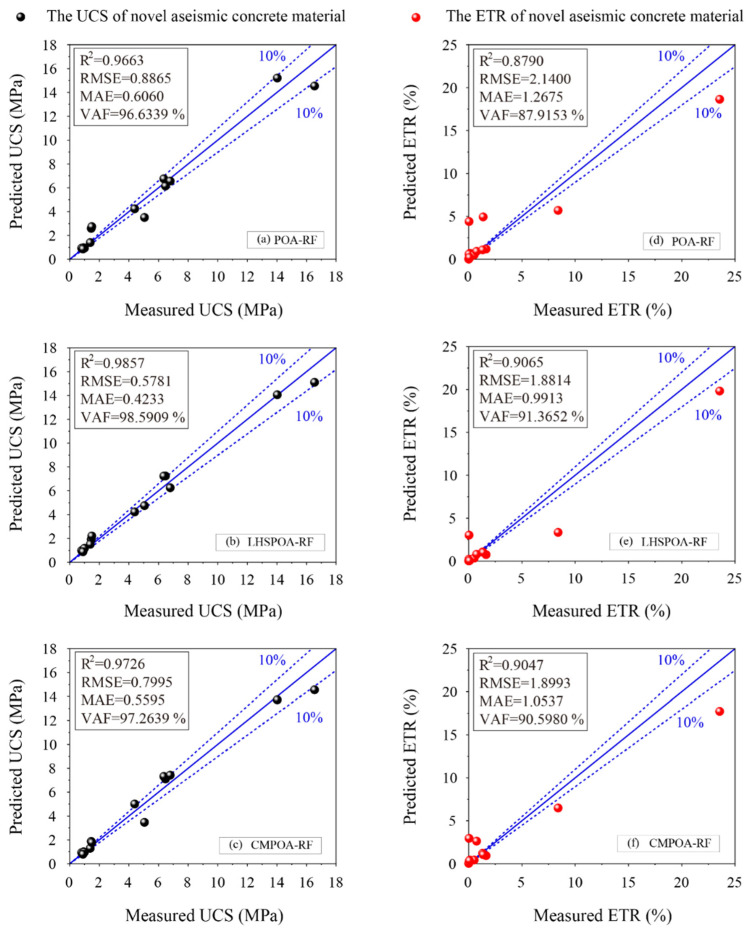
The regression diagrams of the proposed models for predicting the UCS and ETR in the testing phase.

The regression diagrams of the proposed hybrid RF models using the test set are shown in Figure 7. For the UCS prediction, the LHSPOA-RF model has achieved the most satisfactory performance by means of the most points located on the perfect line or closer to it than other models as illustrated in Figure 7b. As shown in Figure 7a,c, the predictive performance of CMPOA-RF is still better than the POA-RF by means of fewer points out of the 10% line. On the other hand, the predictive performance of the proposed models for forecasting the ETR is obviously worse than that of the UCS as shown in Figure 7d,e. However, the regression results showed that the LHSPOA-RF model and the CMPOA-RF model still have better performance than the POA-RF model resulting in the superior performance indices and more effective points close to the perfect line, especially the LHSPOA-RF model. As a results, two improved POA-RF models (i.e., the LHSPOA-RF model and the CMPOA-RF) are considered to be the superior predictors, rather than the initial POA-RF model, in predicting the UCS and the ETR of the novel aseismic rubber-concrete material in this study.

**Figure 8:**
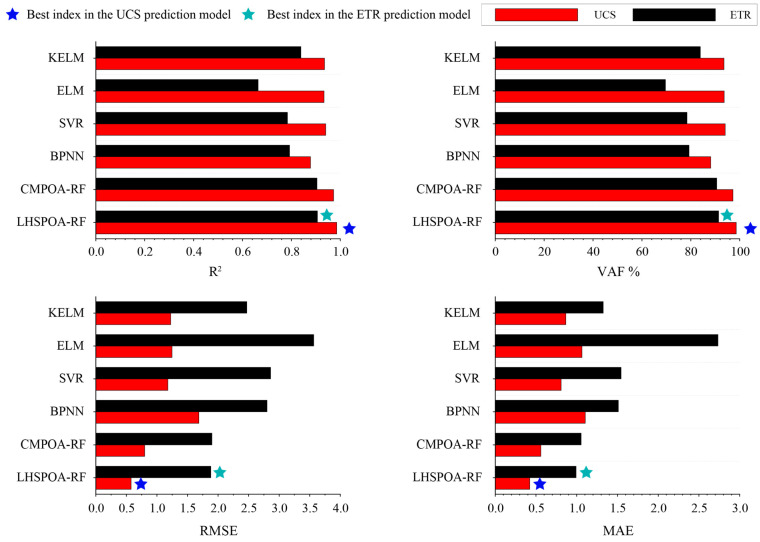
The performance comparison of the proposed models and four common ML models in the UCS and the ETR prediction.

Moreover, four common ML models named the BPNN, support vector regression (SVR), ELM and kernel-extreme learning machine (KELM) are developed to predict the UCS and the ETR of the novel aseismic rubber-concrete material and compare the predictive performances with the proposed models. For the BPNN model, the hyperparameters are the number of hidden layers (N_h_) and the number of neurons in each hidden layer (N_e_). The penalty parameter (P_c_) and the RBF kernel parameter (k_1_) are the main parameters affecting the SVR model performance. The ELM model is a special ANN model with a single hidden layer, thus the number of neurons in this layer (N_n_) can control the performance of the ELM model. The KELM model has similar hyperparameters with the SVR model, i.e., regularization coefficient (R_c_) and the RBF kernel parameter (k_2_). After developing these four ML models, the performance indices of each model with the best hyperparameter combination are shown in Table 8. The performance comparison results of the proposed models and four common ML models in the UCS and the ETR prediction are shown in Figure 8. As can be seen in these pictures, the performance indices of the LHSPOA-RF model and the CMPOA-RF model are obviously better than that of the four ML models, i.e., higher values of R^2^ and VAF, and lower values of RMSE and MAE. Meanwhile, it is obvious that the LHSPOA-RF model is the best model to predict the UCS and the ETR of the novel aseismic rubber-concrete material in this study.

**Figure 9:**
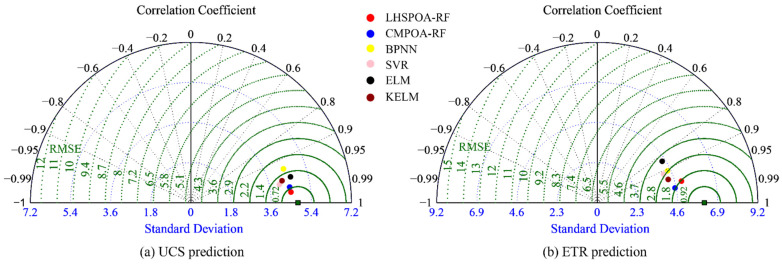
The Taylor diagrams of all models for predicting the UCS and ETR of novel material.

As illustrated in Figure 9, the Taylor diagram is used to make a clear comparison between the proposed models and the four common ML models. This diagram consists of the RMSE, the St. D and the R calculated from the predicted values. In particular, the measured value is fixed on the horizontal axis since its R is equal to 1 and the RMSE is equal to 0. Assuming that the prediction model achieves the best performance, its position on the Taylor diagram is the closest to the measured value. Based on this criterion, the predictive performance of the proposed models is obviously superior to the other four ML models. The LHSPOA-RF is the best prediction model for predicting the UCS and the ETR of this novel aseismic material.

### 6.3. Sensitively Analysis

**Figure 10:**
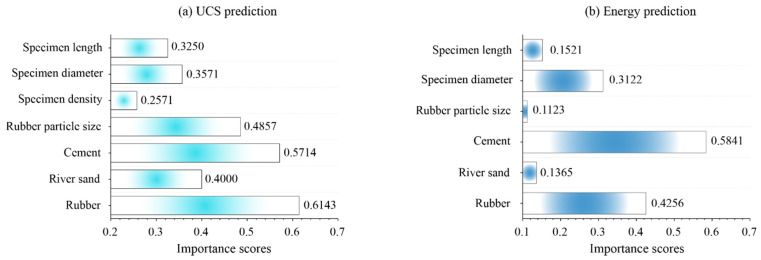
The illustration of sensitive analysis results based the LHSPOA-RF model.

Although the best prediction model has been determined through performance evaluation, the input parameters’ importance for predicting the UCS and the ETR is unknown. Therefore, the PAWN method proposed by Pianosi and Wagener [63,64] is utilized to conduct sensitive analysis based on the LHSPOA-RF model. The parameter importance results are shown in Figure 10. As demonstrated in Figure 10a, rubber is the most important input parameter with a score of 0.6143 for predicting the UCS of the novel aseismic rubber-concrete material. After this parameter, cement (0.5714), rubber particle size (0.4857) and river sand (0.4009) have higher importance scores than specimen diameter (0.3571), specimen length (0.3250) and specimen density (0.2571). For the ETR prediction (see Figure 10b), the cement has the highest importance score of 0.5841 among all the input parameters. The values of rubber and specimen diameter were significantly higher than the other parameters, i.e., rubber (0.4256), specimen diameter (0.3122), specimen length (0.1521), river sand (0.1365) and rubber particle size (0.1123).

## 7. Conclusions

The strength and energy absorption properties of aseismic concrete materials have a significance effect on tunnel stability when a tunnel is shocked by an earthquake and other dynamic impacts. To clearly investigate the above two aseismic performance indices of a novel aseismic concrete material, experimental tests and soft computation techniques are combined in this study. Based on the uniaxial compression test and impact tests, an improved pelican optimization algorithm (POA) using the Latin hypercube sampling (LHS) method and the Chaotic mapping (CM) method was proposed to optimize the random forest (RF) model for predicting the aseismic performance of this novel material. The prediction results demonstrated that the LHSPOA-RF model and CMPOA-RF model have better predictive performances than the general POA-RF model for predicting the UCS and the ETR, especially the LHAPOA-RF model with a more satisfying performance accuracy of R^2^, RMSE, MAE and VAF (UCS: 0.9857, 0.5781, 0.4233 and 98.5909%; ETR: 0.9065, 1.8814, 0.9913 and 91.3652%). Furthermore, the comparison results of four common ML models and the proposed hybrid RF models showed that the LHSPOA-RF model is the best prediction performance among all models.

In addition, rubber plays an important role in strength prediction and cement is the most important parameter for predicting the energy absorption property.

This work indicates that the application of the intelligence models to the aseismic material performance is an effective and more simple method that does not lose accuracy compared with traditional experiment tests in the laboratory. However, the limitation of this combined method is the number of samples in the used database. More and more tested or monitored data should be added into the intelligence models’ development to improve the prediction accuracy in the further research.

## Figures and Tables

**Table 1:** A brief description of the preparation procedure for novel concrete specimen.

Procedures	Description
Step 1—Dosing	Mixing the rubber and river sand with the cement in pre-designed proportions and thoroughly stirring.
Step 2—Concreting	Stirring the mixture for five minutes and quickly pouring it into the mold.
Step 3—Demolding	After 24 h, separating the specimen and polishing it to the specified specification.
Step 4—Maintaining	Maintaining all specimens at required temperature (20 degrees) and humidity (95 %).
Step 5—Testing	After 28 days, testing 140 specimens in the laboratory.

**Table 2:** Mix proportions of the rubber-concrete material.

RM (%)	SM (%)	CM (%)	RPS (mm)
0	100	40	/
10	90	30	1~2
10	90	40	0.5~1
10	90	40	0.075~0.25
10	90	50	0.25~0.5
10	90	60	0.075~0.25
30	70	30	0.5~1
30	70	40	1~2
30	70	40	0.075~0.25
30	70	50	0.075~0.25
30	70	60	0.25~0.5
50	50	30	0.25~0.5
50	50	30	0.075~0.25
50	50	40	1~2
50	50	40	0.5~1
50	50	40	0.25~0.5
50	50	40	0.075~0.25
50	50	50	1~2
50	50	50	0.075~0.25
50	50	60	0.5~1
50	50	60	0.075~0.25
70	30	30	0.075~0.25
70	30	40	0.25~0.5
70	30	40	0.075~0.25
70	30	50	0.5~1
70	30	60	1~2
100	0	40	0.075~0.25

**Table 3:** The detailed description of all considered parameters for the UCS prediction.

Variables	Sign	Unit	Min	Max	Mean	Median	St. D
Rubber	R	g	15.66	90.83	61.21	65.85	22.76
River sand	S	g	33.26	207.63	92.82	74.99	50.52
Cement	C	g	50.52	236.10	129.47	129.38	51.26
Rubber particle size	RPS	mm	0.16	1.50	0.56	0.38	0.48
Specimen mass	M	g	168.40	393.50	283.49	279.25	65.71
Specimen density	r	g/cm^3^	0.98	50.59	16.09	1.72	22.42
Specimen diameter	D	mm	48.99	50.59	50.11	50.15	0.29
Specimen length	L	mm	95.52	102.67	99.05	99.27	1.20
Uniaxial compressive strength	UCS	MPa	0.47	18.52	5.82	4.11	5.02

Note: Min—Minimum value; Max—Maximum value; St. D—Standard deviation value.

**Table 4:** The detailed description of all considered parameters for the ETR prediction.

Variables	Sign	Unit	Min	Max	Mean	Median	St. D
Rubber	R	g	7.28	42.53	29.11	30.38	10.91
River sand	S	g	16.60	97.96	44.35	36.90	23.64
Cement	C	g	24.36	111.48	62.84	60.59	23.65
Rubber particle size	RPS	mm	0.16	1.50	0.56	0.38	0.48
Specimen mass	M	g	81.20	186.70	136.29	138.00	29.59
Specimen density	r	g/cm^3^	0.91	1.95	1.45	1.46	0.30
Specimen diameter	D	mm	48.47	49.98	49.48	49.48	0.30
Specimen length	L	mm	46.46	50.15	48.74	48.86	0.67
Energy transmission rate	ETR	%	0.00	36.43	2.32	0.13	6.37

Note: Min—Minimum value; Max—Maximum value; St. D—Standard deviation value.

**Table 5:** The fitness results of the proposed models based on the different population.

Population	Fitness (RMSE)					
	UCS			ETR		
	POA-RF	LHSPOA-RF	CMPOA-RF	POA-RF	LHSPOA-RF	CMPOA-RF
20	0.07503	0.06054	0.06338	0.16103	0.13669	0.14078
40	0.08197	0.06505	0.06265	0.16081	0.13484	0.14024
60	0.07419	0.06329	0.06277	0.16199	0.13340	0.14158
80	0.08340	0.05929	0.06154	0.16085	0.13294	0.14239
100	0.08368	0.06324	0.06159	0.16083	0.13671	0.14238
Best hyperparameters combination						
N_t_	15	22	17	18	13	14
Maxdepth	2	2	2	1	1	1

**Table 6:** The performance indices of the proposed models in the training phase.

**Models**	**UCS Prediction**							
	**Performance Indices**							
	**R^2^**	**Score**	**RMSE**	**Score**	**MAE**	**Score**	**VAF (%)**	**Score**
POA-RF	0.9654	1	0.9285	1	0.5913	1	96.5393	1
LHSPOA-RF	0.9800	3	0.7057	3	0.4461	3	98.0005	3
CMPOA-RF	0.9761	2	0.7710	2	0.4652	2	97.6378	2
**Models**	**ETR Prediction**							
	**Performance Indices**							
	**R^2^**	**Score**	**RMSE**	**Score**	**MAE**	**Score**	**VAF (%)**	**Score**
POA-RF	0.8814	1	2.2052	1	0.8571	1	88.1907	1
LHSPOA-RF	0.9108	3	1.9128	3	0.7364	3	91.0880	3
CMPOA-RF	0.9062	2	1.9608	2	0.7732	2	90.6635	2

**Table 7:** The performance indices of the proposed models in the testing phase.

**Models**	**UCS Prediction**							
	**Performance Indices**							
	**R^2^**	**Score**	**RMSE**	**Score**	**MAE**	**Score**	**VAF (%)**	**Score**
POA-RF	0.9663	1	0.8865	1	0.6060	1	96.6339	1
LHSPOA-RF	0.9857	3	0.5781	3	0.4233	3	98.5909	3
CMPOA-RF	0.9726	2	0.7995	2	0.5595	2	97.2639	2
**Models**	**ETR Prediction**							
	**Performance Indices**							
	**R^2^**	**Score**	**RMSE**	**Score**	**MAE**	**Score**	**VAF (%)**	**Score**
POA-RF	0.8790	1	2.1400	1	1.2675	1	87.9153	1
LHSPOA-RF	0.9065	3	1.8814	3	0.9913	3	91.3652	3
CMPOA-RF	0.9047	2	1.8993	2	1.0537	2	90.5980	2

**Table 8:** The performance of the other four AI models using test set.

**Models**	**UCS Prediction**				**Hyperparameter**
	**Performance Indices**				
	**R^2^**	**RMSE**	**MAE**	**VAF (%)**	
BPNN	0.8782	1.6859	1.1049	88.1409	N_h_ = 1; N_e_ = 8
SVR	0.9406	1.1779	0.8091	94.0956	P_c_ = 64; k_1_ = 0.5
ELM	0.9334	1.2464	1.0643	93.6570	N_n_ = 40
KELM	0.9356	1.2257	0.8654	93.5694	R_c_ = 32; k_2_ = 0.5
**Models**	**ETR Prediction**				**Hyperparameter**
	**Performance Indices**				
	**R^2^**	**RMSE**	**MAE**	**VAF (%)**	
BPNN	0.7926	2.8016	1.5106	79.2861	N_h_ = 1; N_e_ = 6
SVR	0.7838	2.8604	1.5438	78.4293	P_c_ = 35; k_1_ = 0.25
ELM	0.6641	3.5650	2.7334	69.6411	N_n_ = 60
KELM	0.8388	2.4700	1.3255	83.8904	R_c_ = 55; k_2_ = 0.15

## Data Availability

The data used in this article is confidential.
